# Influence of Methyl Jasmonate and Short-Term Water Deficit on Growth, Redox System, Proline and Wheat Germ Agglutinin Contents of Roots of Wheat Seedlings

**DOI:** 10.3390/ijms26146871

**Published:** 2025-07-17

**Authors:** Alsu R. Lubyanova

**Affiliations:** Institute of Biochemistry and Genetics—Subdivision of the Ufa Federal Research Centre of the Russian Academy of Sciences, Prospect Oktyabrya 71, Lit. 1e, 450054 Ufa, Russia; molgen@anrb.ru or lubyanova555@mail.ru; Tel.: +7-987-097-4257

**Keywords:** catalase, hydrogen peroxide, methyl jasmonate, peroxidase, polyethylene glycol 6000, proline, superoxide dismutase, superoxide radical, wheat germ agglutinin, *Triticum aestivum* L.

## Abstract

Drought is a serious environmental problem that limits the yield of wheat around the world. Using biochemical and microscopy methods, it was shown that methyl jasmonate (MeJA) has the ability to induce the oxidative stress tolerance in roots of wheat plants due to the regulation of antioxidant enzymes activity, proline (Pro), and wheat germ agglutinin (WGA) accumulation. During the first hours of 12% polyethylene glycol (PEG) exposure, stress increased the superoxide radical (O_2_^•−^) and the hydrogen peroxide (H_2_O_2_) accumulation, the activity of superoxide dismutase (SOD), total peroxidase (POD), ascorbate peroxidase (APX), catalase (CAT), the percent of dead cells (PDC), malondialdehyde accumulation (MDA), and electrolyte leakage (EL) of wheat roots as compared to the control. Stress enhanced proline (Pro) and wheat germ agglutinin (WGA) contents in roots and the plant’s nutrient medium, as well as decreased the mitotic index (MI) of cells of root tips in comparison to the control. During PEG exposure, 10^−7^ M MeJA pretreatment increased the parameter of MI, declined O_2_^•−^ and H_2_O_2_ generation, PDC, MDA, and EL parameters as compared to MeJA-untreated stressed seedlings. During 1 day of drought, MeJA pretreatment additionally increased the activity of SOD, total POD, APX, CAT, Pro, and WGA accumulation in wheat roots in comparison to MeJA-untreated stressed plants. During stress, MeJA pretreatment caused a decrease in Pro exudation into the growth medium, while WGA content in the medium was at the control level.

## 1. Introduction

A wide range of abiotic stresses, such as water deficit, soil salinity, extreme temperatures, flooding, exposure to excess ions, heavy metals, and irrational use of xenobiotic compounds, are induced in plant organisms, such as secondary stresses as osmotic and oxidative ones [1,2]. It should be noted that most of the global cultivation area is exposed to one or another kind of stress [2,3], and drought is one of the major crop yield and quality limiting stress factor throughout the world [4,5,6]. More than half of the irrigated agricultural land areas are known to be affected by prolonged or frequent drought [7].

The global food demand is growing due to the increase in human population and depletion of agricultural lands [4,8]. Among crops, bread wheat (*Triticum aestivum* L.) is the most common and important cereal plant [4]. To date, studies of the cellular and molecular mechanisms of plant development and their response to environmental stresses are being actively carried out on dicotyledonous plants, while the main agricultural crops are monocotyledonous plants, which may have differences in adaptive strategies compared to dicots. For example, the exogenous proline (Pro) treatment induced the opposite growth response of tobacco and rice seedlings [9].

Due to its primary absorptive function, the plant root system is the first site to perceive an oncoming drought and adapt to the maximum possible water and nutrient intake under water deficit conditions, so the root traits should be carefully examined in research and selection programs [10]. The root system plays a vital role in plant growth, development of overall productivity, and survival [4], providing attachment to the substrate, the entrance of water and nutrients dissolved in soil into the plant organism, the synthesis and transport of specific signaling molecules from the roots to the shoots and out to the environment [11]. Compared with shallow root system architecture, deep-rooted plants have larger-sized grains, higher grain weight, and yield [12]. Moreover, the increased parameters of antioxidant capacity and proline content correspond to tolerant wheat genotypes [5]. The different plant tissues unequally respond to environmental influences and treatments [13,14], the roots’ response to drought can differ from that of the whole plant.

In recent years, the efforts of researchers have focused on developing stress-tolerant plants and attaining their high yields on existing croplands [8]. The approach to treat the seeds or plants with phytohormone-based preparations in order to increase stress resistance and yield is already well established [15,16], especially for potted plants, but is not widely used in the production of cereals due to their high cost. Biostimulants may contain phytohormones or hormone-like substances, and they can improve plant growth, crop yield, product quality, and tolerance to abiotic stress [17,18]. To date, the promising direction is the investigation of the effects of phytohormones on the physiology and biochemistry of plants to obtain new varieties by genetic engineering methods in the future [18].

Phytohormones are present in plant organisms at extremely low concentrations as key regulators of a plant’s metabolism. One of the important groups of plant hormones is jasmonates, which include jasmonic acid (JA) and its derivatives: methyl jasmonate (MeJA), 3R,7S-jasmonoyl-L-isoleucine (JA-Ile), *cis*-jasmonate, and jasmonyl 1-aminocyclopropane-1-carboxylic acid (JA-ACC) [19,20,21]. Deficiencies in the biosynthesis or perception of the jasmonates impair stamen development, male fertility, and stop seed production in *Arabidopsis thaliana* [22]. Jasmonates are often used for post-harvest processing of fruits and berries in order to increase their shelf life and quality [15,23,24]. The MeJA application can be useful for enhancing secondary metabolite production in plant culture systems in vitro [20]. Even a short-term exogenous treatment of plants with jasmonates induces adventitious root formation [25]. During stresses, jasmonates regulate the competitive distribution of energy [16], increase resistance [21,26,27], and expression of stress-induced genes [20,26,28]. JA-induced drought tolerance in plants is implemented through closing stomata, controlling reactive oxygen species (ROS) generation, and deeper root growth [18]. However, the data on the jasmonates’ influence on plant growth are contradictory [16]. It is known that jasmonate-induced inhibition [29,30] and stimulation of plant growth [31,32,33,34,35]. This fact may be due to the involvement of jasmonates and other phytohormones, signaling molecules in a complex network of coordination of growth and development of sessile plants under stressful environmental conditions [3,20,21,23,36,37], as important physiological responses are usually duplicated by different signaling pathways. For example, signaling of both ABA and jasmonates induces stomatal closure [38], drought tolerance [39], and JA may act even earlier than ABA.

It is known that stress-induced rapid ROS production can induce JA synthesis [40] and activate JA-related signaling pathways [39,41]. In turn, an increase in the level of jasmonates in tissues enhances the antioxidant enzymatic and non-enzymatic activity of plants [15,23,24]. Jasmonates treatment stimulates the activity of enzymatic antioxidants superoxide dismutase (SOD), peroxidase (POD), and catalase (CAT) [28,42] and reduces stress-induced oxidative burst [43,44]. Salinity-induced ROS accumulation damaged JA-deficient *def-1* mutants of tomato plants (*Solanum lycopersicum* L.) due to a decrease in the activity of both enzymatic and non-enzymatic antioxidants [32]. The influence on the activity of ROS-scavenging enzymes is dependent on jasmonate’s concentration; their high concentrations could have an inhibitory effect [28].

Proteomic investigations have contributed to our understanding of the mechanisms underpinning the activity of different plant biostimulants, including phytohormones, under normal and challenging growth conditions. Lectins are fundamental proteins for plants; they play an important role in cell-to-cell communication, developmental strategy, and environmental stress resistance [45]. Wheat germ agglutinin (WGA) is a characteristic protein of wheat plants. It can interact with *N*-acetyl-*D*-glucosamine [46] and phytohormones [47], probably regulating their amount and/or activity. WGA affects the growth and hormonal status of wheat and closely related monocotyledonous species [48]. Like strigolactones and auxins [49], WGA is a root-excreted protein, and it can be assumed that in this way it regulates the growth and/or stress resistance of neighboring plants. For example, WGA pretreatment reduces the level of oxidative stress in wheat plants under salinity [50].

The coordinated interaction and regulation of antioxidant enzymes activity and accumulation of ROS, Pro, and other plant metabolites for improved stress tolerance and increased crop yield under challenging environmental influences are necessary. The purpose of this investigation was to elucidate the effect of MeJA pretreatment on the development of oxidative stress and activity of the enzymatic antioxidants of wheat roots under short-term water deficit. In addition, this study examined how MeJA affected the root growth, the death of root cells, accumulation and excretion of such putative antioxidants as amino acid Pro and carbohydrate-binding protein WGA in roots of wheat seedlings, subjected to short-term 12% PEG exposure.

## 2. Results

### 2.1. The Influence of 10^−7^ M MeJA Application on ROS Generation

**Figure 1:**
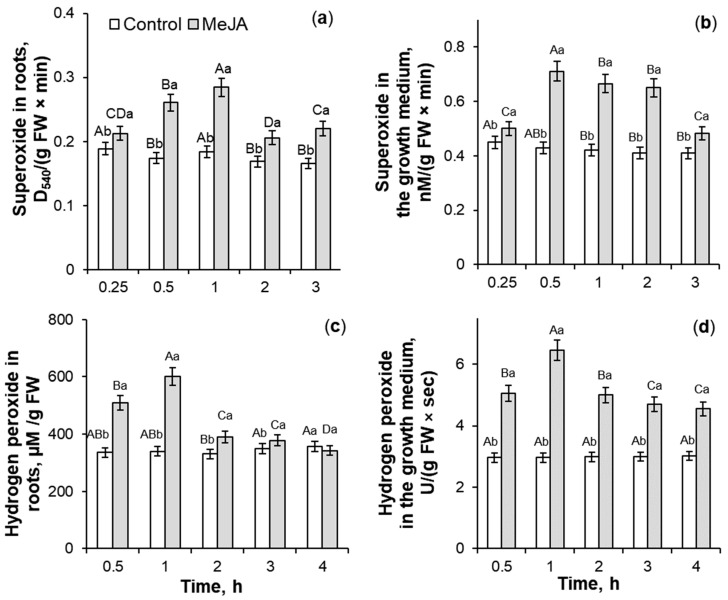
The effect of exogenous 10^−7^ M MeJA treatment on superoxide radical accumulation in roots (**a**), exudation of O_2_^•−^ (**b**), accumulation of hydrogen peroxide in roots (**c**), and H_2_O_2_ excretion into the growth medium (**d**) of 6-day-old wheat plants. Data are given as mean values and their standard errors from three biological and four analytical repeats. Lowercase letters above the columns indicate significant differences between variants of treatment at the particular time point at *p* < 0.05 (ANOVA, LSD test). Capital letters indicate significant differences for the same treatment at different time points at *p* < 0.05 (ANOVA, LSD test). FW—fresh weight; MeJA—methyl jasmonate.

Treatment with 10^−7^ M MeJA for 0.25–3 h induced a 1.1–1.6-fold and 1.1–1.8-fold increase of O_2_^•−^ accumulation in roots and the plant’s nutrition medium, respectively, as compared to control seedlings (Figure 1a,b). The maximum of the superoxide anion generation was at 0.5–1 h of the phytohormone treatment. The MeJA application of wheat seedlings for 0.5–4 h caused a 1.1–1.8-fold induction of H_2_O_2_ generation in roots and a 1.5–2.2-fold increase of hydrogen peroxide accumulation in Hoagland–Arnon solution as compared to the control (Figure 1c,d). The maximum increase in hydrogen peroxide accumulation was observed at 1 h of MeJA exposure. So, the process of MeJA-induced ROS accumulation in root tissues is accompanied by the stimulation of their release into the growth medium of wheat plants.

### 2.2. Antioxidant Enzymes Activity Under 10^−7^ M MeJA Application

**Figure 2:**
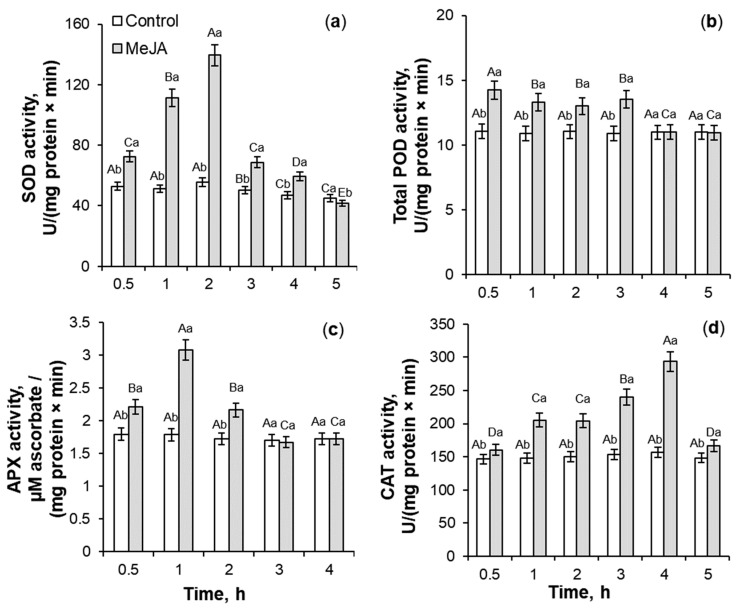
The influence of exogenous 10^−7^ M MeJA treatment for 0.5–5 h on superoxide dismutase (SOD, (**a**)), total peroxidase (POD, (**b**)), ascorbate peroxidase (APX, (**c**)), and catalase (CAT, (**d**)) activities in roots of 6-day-old wheat. Data are given as mean values and their standard errors from three biological and four analytical repeats. Lowercase letters above the columns indicate significant differences between variants of treatment at the particular time point at *p* < 0.05 (ANOVA, LSD test). Capital letters indicate significant differences for the same treatment at different time points at *p* < 0.05 (ANOVA, LSD test).

With the magnification in the ROS accumulation (Figure 1), the activity of SOD, total POD, ascorbate peroxidase (APX), and CAT in roots of MeJA-treated wheat seedlings showed a transient increase (Figure 2). MeJA treatment for 0.5–5 h resulted in more than a 2-fold increase in SOD activity as compared to the control roots. The phytohormone application induced an increase in total POD and CAT activity by 18–29% and 10–90%, respectively, relative to the control plants (Figure 2b,d). The highest SOD and CAT activity was induced for 2 and 4 h of MeJA application, respectively (Figure 2a,d), while the induction of total POD activity was registered for the first 0.5–3 h of MeJA treatment (Figure 2b) as compared to MeJA-untreated wheat roots. APX activity showed a 1.7-fold increase during 3 h of phytohormone influence, with a maximum at 1 h of MeJA treatment (Figure 2c).

**Figure 3:**
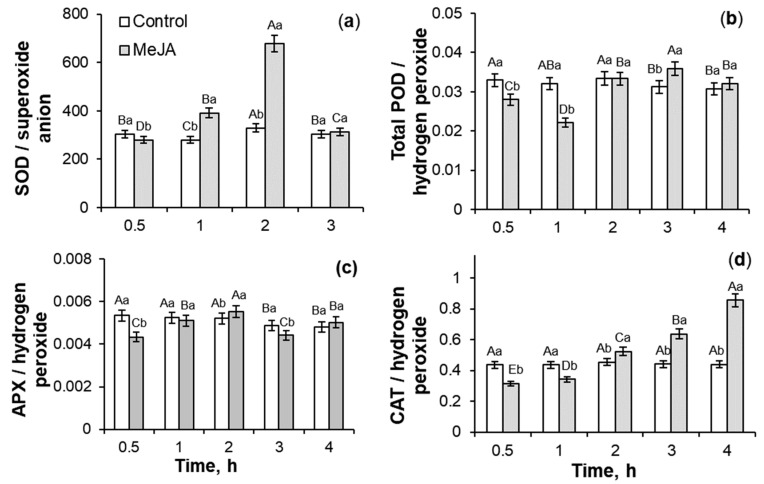
The effect of exogenous 10^−7^ M MeJA treatment on coefficients of SOD/superoxide radical (**a**), total POD/hydrogen peroxide (**b**), APX/H_2_O_2_ (**c**), and CAT/hydrogen peroxide (**d**) of roots of 6-day-old wheat plants. Data are given as mean values and their standard errors. Lowercase letters above the columns indicate significant differences between variants of treatment at the particular time point at *p* < 0.05 (ANOVA, LSD test). Capital letters indicate significant differences for the same treatment at different time points at *p* < 0.05 (ANOVA, LSD test).

The analysis revealed that under MeJA treatment, the coefficients of peroxidase activities/hydrogen peroxide didn’t increase significantly, and during 0.5–1 h of MeJA application, even decreased as compared to the control plants (Figure 3b,c). MeJA treatment for 1–2 h induced a transient 1.4–2.1-fold increase in the coefficients of SOD activity/superoxide anion in comparison to control (Figure 3a). The MeJA application for 2–4 h stimulated a gradual and significant 1.2–2-fold increase in the ratio of CAT activity/hydrogen peroxide as compared to control seedlings (Figure 3d).

### 2.3. The Effect of MeJA Pretreatment on Mitotic Index and Percent of Dead Cells Under 12% PEG Exposure

**Figure 4:**
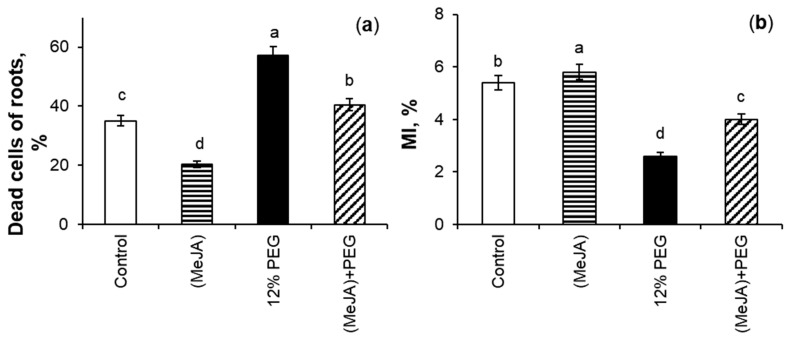
The influence of exogenous 10^−7^ M MeJA pretreatment for 24 h followed by 12% PEG exposure for 24 h on the percentage of dead cells (**a**) and mitotic index of cells (MI, (**b**)) of roots of 8-day-old wheat plants. Values are the mean of three replicates ± SE. To assess the level of PDC *n* = 10, to estimate the level of MI, *n* = 1500. Means are considered different at *p* ≤ 0.05 when they are followed by different letters within the same parameter.

Figure 4a describes the impact of MeJA pretreatment on the percentage of dead cells (PDC) of roots of wheat seedlings both under water deficit and non-stressful conditions. MeJA pretreatment promoted a 1.7-fold reduction in the PDC parameter of wheat roots as compared to the control. The highest PDC was obtained under drought; this parameter increased 1.6-fold relative to the control plants. The level of dead cells in the MeJA-pretreated roots under 12% PEG exposure was 1.4-fold lower than under stress alone, and 1.2-fold higher than compared to control seedlings.

The effect of MeJA pretreatment under drought stress on wheat root growth was investigated using light microscopy analysis as a percentage of dividing cells of the root tips (Figure 4b). The control and MeJA-pretreated plants had the highest parameters of mitotic index (MI). The MeJA application increased the level of MI by 7% as compared to the control. 12% PEG exposure showed a 2.1-fold decrease in MI in comparison to the control seedlings. During drought for 24 h, MeJA-pretreated wheat seedlings increased the MI parameter by 54% as compared to MeJA-untreated stressed plants.

### 2.4. The Effect of MeJA Pretreatment on ROS Generation in Wheat Roots Under 12% PEG Exposure

**Figure 5:**
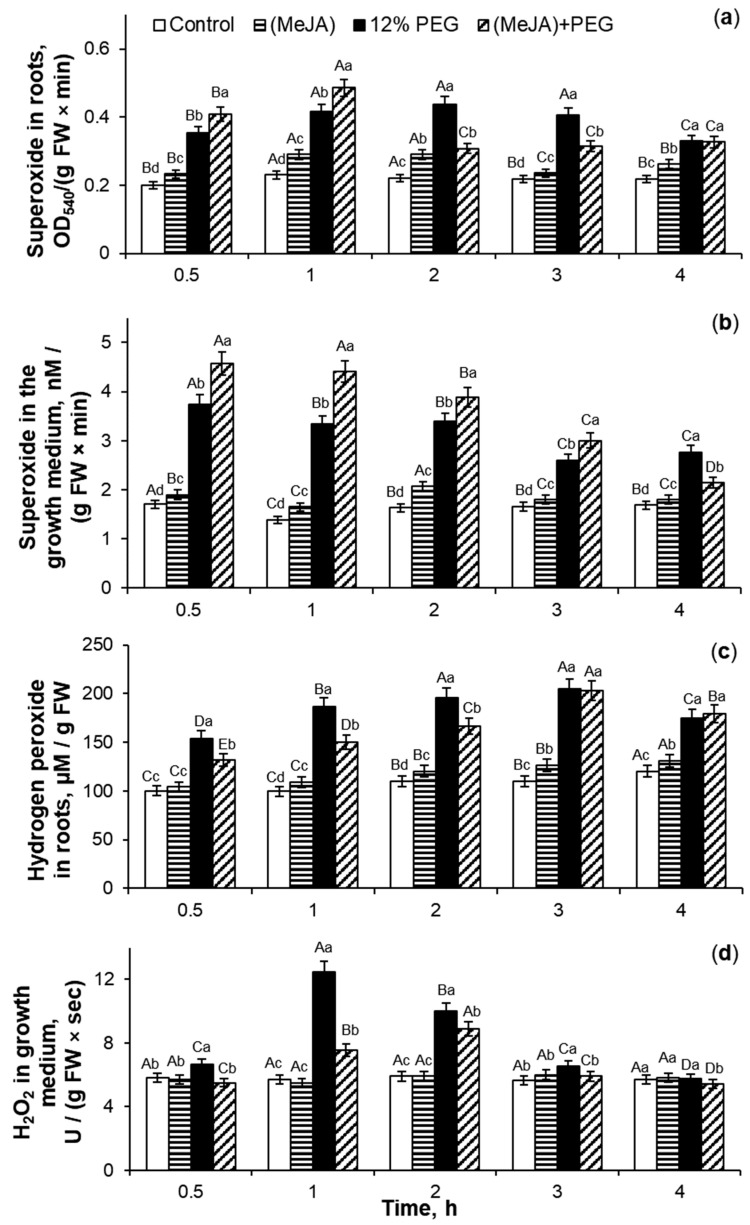
The effect of 10^−7^ M MeJA pretreatment for 24 h followed by 12% PEG exposure for 0.5–4 h on superoxide anion accumulation in wheat roots (**a**) and in the growth medium (**b**), hydrogen peroxide generation in the roots (**c**) and H_2_O_2_ accumulation in the growth medium (**d**) of 7-day-old wheat plants. Data are given as mean values and their standard errors from three biological and four analytical repeats. Lowercase letters above the columns indicate significant differences between variants of treatment at the particular time point at *p* < 0.05 (ANOVA, LSD test). Capital letters indicate significant differences for the same treatment at different time points at *p* < 0.05 (ANOVA, LSD test).

After cessation of MeJA exposure, superoxide anion generation in wheat roots (Figure 5a) and its accumulation in Hoagland–Arnon medium (Figure 5b) increased by 7–31% and 7–27%, respectively, as compared to control. The ROS production significantly increased in PEG-treated roots of wheat seedlings as compared to control plants. Drought stress for 4 h caused 1.4–2-fold increase in superoxide anion generation in roots and 1.6–2.4-fold enhancement of O_2_^•−^ accumulation in the Hoagland–Arnon medium relative to control. During PEG exposure for 0.5–1 h, MeJA-pretreated wheat roots generated O_2_^•−^ by 16–17% more than MeJA-untreated stressed plants (Figure 5a). During further subjection of 12% PEG for 2–3 h, the superoxide accumulation in MeJA-pretreated wheat roots decreased by 22–30% as compared to MeJA-untreated stressed seedlings. Under stress influence for 3 h, MeJA-pretreated wheat roots released O_2_^•−^ into the growth medium by 22–39% more than MeJA-untreated stressed plants (Figure 5b). Under PEG exposure for 4 h, the level of superoxide anion in surrounding medium of MeJA-pretreated stressed wheat roots decreased by 22% as compared to MeJA-untreated stressed seedlings.

MeJA application induced an increase of hydrogen peroxide generation in wheat roots by 4–15% and did not change H_2_O_2_ accumulation in the growth medium (Figure 5c,d) as compared to control seedlings. Exposure to 12% PEG induced a 1.5–1.9-fold increase of hydrogen peroxide generation in roots and 1.7–2.2-fold enhancement of H_2_O_2_ accumulation in the growth medium in comparison to the control. After MeJA pretreatment, the effect of drought on hydrogen peroxide production of roots of wheat seedlings was significantly lower compared to the MeJA-untreated stressed plants. MeJA-pretreated stressed wheat roots increased the level of hydrogen peroxide generation by 25–56% relative to control (Figure 5c). Under drought conditions, the MeJA application decreased H_2_O_2_ accumulation in the growth medium of wheat seedlings by 6–40% as compared to MeJA-untreated stressed plants (Figure 5d).

### 2.5. MeJA-Induced Regulation of Activity of Antioxidant Enzymes During Drought Stress

**Figure 6:**
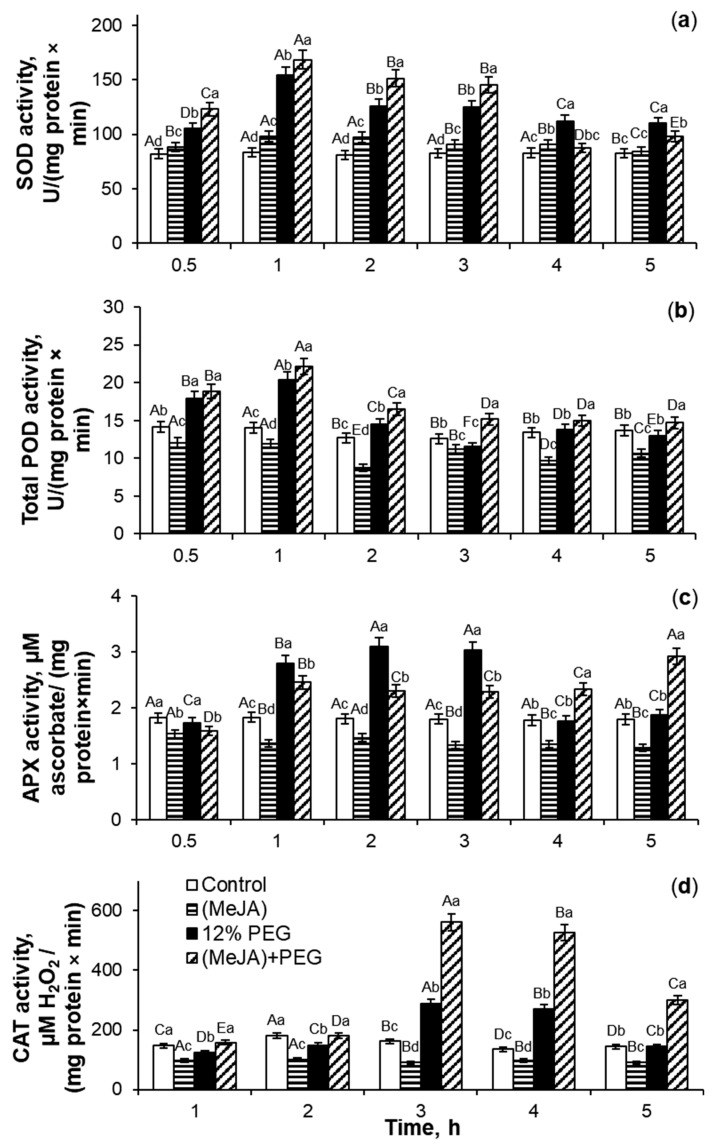
The influence of 10^−7^ M MeJA pretreatment for 24 h followed by 12% PEG exposure for 0.5–5 h on superoxide dismutase (SOD, (**a**)), total peroxidase (POD, (**b**)), ascorbate peroxidase (APX, (**c**)), and catalase (CAT, (**d**)) activities in roots of 7-day-old wheat seedlings. Data are given as mean values and their standard errors from three biological and four analytical repeats. Lowercase letters above the columns indicate significant differences between variants of treatment at a particular time point at *p* < 0.05 (ANOVA, LSD test). Capital letters indicate significant differences for the same treatment at different time points at *p* < 0.05 (ANOVA, LSD test).

Under non-stressful conditions, MeJA pretreatment induced a slight increase in SOD activity and significantly decreased the activities of total POD, APX, and CAT in roots of wheat seedlings as compared to the control (Figure 6a).

Exposure to 12% PEG for 0.5–5 h led to an increase in SOD activity by 28–85%, with a maximum at 1 h of stress influence relative to the control plants. During 0.5–3 h of PEG exposure, MeJA pretreatment increased SOD activity by 9–20%, while after 4–5 h of stress exposure, MeJA declined this parameter by 10–21% as compared to MeJA-untreated stressed seedlings.

The MeJA application decreased total POD activity in roots of wheat seedlings by 11–31% as compared to the control (Figure 6b). Drought induced the enhancement of total POD activity in wheat roots by 7–58% relative to the control. Under PEG exposure, MeJA pretreatment increased the total POD activity of roots of wheat seedlings by 5–13% in comparison to MeJA-untreated stressed plants.

MeJA pretreatment decreased APX activity by 15–28% as compared to the control (Figure 6c). During the first 1–3 h of PEG treatment, water deficit induced an increase in APX activity by 53–72% relative to the control plants. The maximum increase in APX activity was observed at 2 h of PEG exposure. During 0.5–3 h of PEG subjection, MeJA pretreatment decreased APX activity of roots of wheat seedlings, while stress treatment for 4–5 h induced the activation of APX activity of MeJA-pretreated plants by 31–56% as compared to MeJA-untreated stressed seedlings.

MeJA pretreatment decreased CAT activity in wheat roots by 28–46% under non-challenging growth conditions (Figure 6d). Water deficit decreased CAT activity during the first two hours of PEG exposure and induced a 1.8–2.0-fold increase in CAT activity of MeJA-pretreated seedlings after 3–4 h of stress influence as compared to the control plants. Stress did not significantly change the CAT activity of MeJA-pretreated plants during the first two hours of PEG exposure, while PEG subjection for 3–4 h caused a 2.1–3.9-fold increase in CAT activity of wheat roots in comparison to the control.

**Figure 7:**
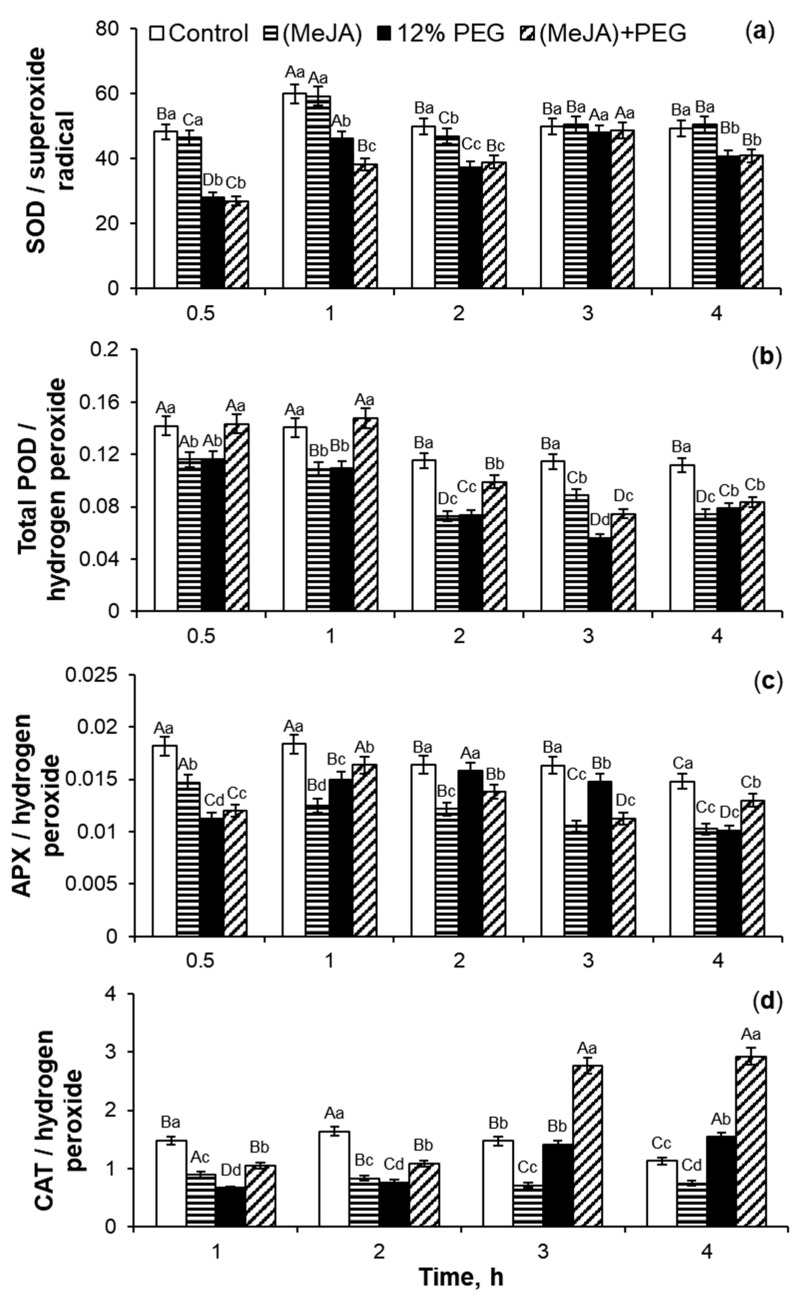
The effect of 10^−7^ M MeJA pretreatment for 24 h followed by 12% PEG exposure for 0.5–4 h on coefficients of SOD activity/superoxide radical (**a**), total POD activity/hydrogen peroxide (**b**), APX activity/hydrogen peroxide (**c**), and CAT activity/hydrogen peroxide (**d**) in roots of 7-day-old wheat seedlings. Data are given as mean values and their standard errors. Lowercase letters above the columns indicate significant differences between variants of treatment at the particular time point at *p* < 0.05 (ANOVA, LSD test). Capital letters indicate significant differences for the same treatment at different time points at *p* < 0.05 (ANOVA, LSD test).

The analysis of the coefficients of antioxidant enzyme activity/ROS (Figure 7) showed that after MeJA cessation the parameter of SOD activity/O_2_^•−^ did not change, but the ratios of activities of oxidoreductases total POD, APX, and CAT in relation to hydrogen peroxide decreased by 18–37%, 18–33%, and 35–49%, respectively, as compared to the control seedlings (Figure 7b–d). 12% PEG exposure during 4 h decreased the coefficient of POD activity/hydrogen peroxide by 17–51% in comparison to the control (Figure 7b). During drought for 1–4 h, the coefficients of SOD activity/superoxide anion and APX activity/hydrogen peroxide fluctuated, but in total, their parameters decreased as compared to the control plants (Figure 7a,c). Under PEG subjection, the trend of CAT activity/hydrogen peroxide coefficients revealed a gradual increase as compared to the control (Figure 7d). Under PEG exposure, MeJA-pretreated wheat seedlings demonstrated the fluctuations in the MeJA-induced regulation of activity of SOD and APX in relation to ROS as compared to MeJA-untreated stressed plants (Figure 7a,c). During 12% PEG influence for 1–2 h, MeJA-pretreated wheat plants showed an increase in the coefficients of CAT activity/hydrogen peroxide by 42–60% as compared to MeJA-untreated stressed seedlings. Drought for 3–4 h caused a 1.9-fold increase in the coefficients of CAT activity/hydrogen peroxide in comparison to MeJA-untreated stressed plants (Figure 7d).

The activity of antioxidant enzymes and ROS accumulation in the roots of wheat seedlings were monitored during 12% PEG exposure for 24 h (Table 1). No significant reduction in ROS accumulation was found in MeJA pretreated roots as compared to the control seedlings (Table 1). These findings correlated with small MeJA-caused induction of SOD activity and a noticeable increase in total POD, APX, and CAT activity in the roots of wheat plants by 11%, 28%, and 50%, respectively, in comparison to the control. Under drought stress, there was a significant 1.6–1.8-fold increase in both superoxide anion and hydrogen peroxide in the roots of wheat seedlings as compared to the control plants. During PEG exposure, the increase of O_2_^•−^ and H_2_O_2_ generation in wheat roots is accompanied by the induction of SOD, total POD, APX, and CAT activity by 34%, 34%, 56%, and 95%, respectively, as compared to the control. Under PEG subjection, MeJA pretreatment increased SOD, total POD, APX, and CAT activity by 11%, 5%, 25%, and 13%, respectively, and decreased O_2_^•−^ and H_2_O_2_ accumulation in wheat roots by 17% and 32%, respectively, in comparison to MeJA-untreated stressed seedlings.

After MeJA cessation, the phytohormone pretreatment increased the coefficients of SOD activity/O_2_^•−^, total POD activity/H_2_O_2_, APX activity/H_2_O_2_, and especially, CAT activity/H_2_O_2_ by 6%, 15%, 13%, and 56%, respectively, as compared to the control (Table 2). 12% PEG exposure during 24 h decreased the coefficients of SOD activity/superoxide anion and total POD activity/hydrogen peroxide by 24% and 14%, respectively, in comparison to control plants, while the coefficient of APX activity/H_2_O_2_ was near the control level. Water deficit for 24 h increased the coefficient of CAT activity/H_2_O_2_ by 25% as compared to the control and decreased it by 20% as compared to MeJA-pretreated plants. Under PEG exposure, MeJA pretreatment induced an additional increase in the ratios of SOD activity/superoxide anion and total POD activity/hydrogen peroxide by 34% and 53%, respectively, as compared to MeJA-untreated stressed plants. Under stress subjection, a significant 1.75-fold and 1.65-fold increase in the coefficients of APX activity/H_2_O_2_ and CAT activity/H_2_O_2_, respectively, were observed in MeJA-pretreated plants in comparison to MeJA-untreated seedlings.

MeJA-pretreated and control roots contained and released into the growth medium small amounts of Pro (Table 3). MeJA pretreatment did not change the Pro level in the roots, but increased Pro excretion by 23% as compared to the control plants. Drought induced a 2-fold and 59-fold accumulation of this amino acid in the roots and the plant’s growth medium, respectively, relative to the control. During 12% PEG exposure, MeJA pretreatment caused additional Pro accumulation in wheat roots by 22% and reduced Pro excretion into the Hoagland–Arnon solution by 27% in comparison to MeJA-untreated stressed wheat seedlings.

Evaluation of the degree of cell damage was accomplished using the electrolyte leakage (EL) and malondialdehyde (MDA) measurements. MeJA pretreatment did not affect the MDA, but increased the EL level by 18% as compared to the control plants (Table 3). Exposure to 12% PEG induced MDA accumulation by 90% and a 4-fold magnification of the EL level of wheat roots in comparison to the control roots. Under PEG influence for 24 h, MeJA pretreatment decreased MDA and EL parameters by 18% and 62%, respectively, as compared to MeJA-untreated stressed plants.

**Figure 8:**
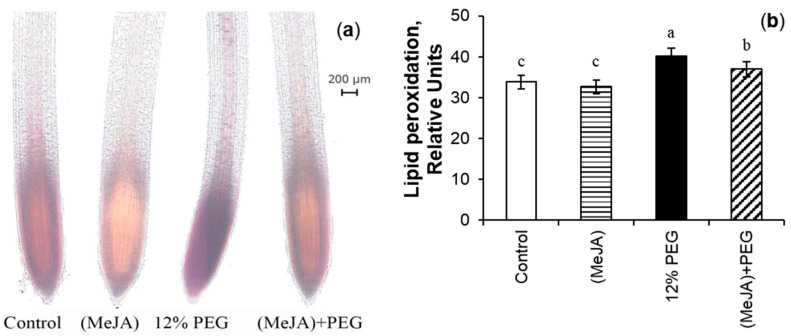
Representative images illustrating in situ histochemical localization of lipid peroxidation (**a**), and relative quantification assay of the products of lipid peroxidation (**b**) in tips of 8-day-old wheat roots after 10^−7^ M MeJA pretreatment for 24 h, followed by 12% PEG exposure for 24 h. Scale bar = 200 µm. Image J software 1.41o/Java 1.6.0_10 (NIH, Bethesda, MD, USA) was used to calculate the relative level of lipid peroxidation by integrated density. Data are the mean ± SE (*n* = 10). Different letters above the columns imply that the values differ significantly at *p* < 0.05 (ANOVA, LSD test).

The histochemical study was performed using light microscopy. The coloring of wheat roots with Schiff’s reagent indicated that the active lipid peroxidation was localized in the root tip and the vascular cylinder (Figure 8a). Even the roots of control plants had the baseline level of lipid peroxidation as their tips (the zone of active cell proliferation and cell expansion), and the vascular cylinder was stained a purple color. MeJA pretreatment decreased lipid peroxidation of roots of wheat plants under non-challenging growth conditions. Exposure to 12% PEG increased the area of staining of the root tip zone. The stress-induced reaction of lipid peroxidation intensified in all plant tissues, especially in zones of the root tip and vascular cylinder, confirming the addition of stress-triggered peroxidation to the physiological one. Under PEG exposure MeJA pretreatment declined lipid peroxidation of wheat roots as compared to MeJA-untreated stressed seedlings.

Ten representative images were quantified by Image J software, revealing differences between MeJA-pretreated and untreated seedlings under drought (Figure 8b). The intensity and area of staining were the same in the roots of the control and MeJA-pretreated wheat roots under non-challenging growth conditions. When analyzed with Image J software, the roots of PEG-treated wheat seedlings showed an increase in lipid peroxidation level by 18% as compared to the control roots. During 12% PEG exposure, the level of lipid peroxidation in MeJA-untreated wheat roots was higher by 7.5% than that of MeJA-pretreated seedlings.

### 2.6. MeJA-Induced Regulation of WGA Accumulation Under 12% PEG Treatment

**Figure 9:**
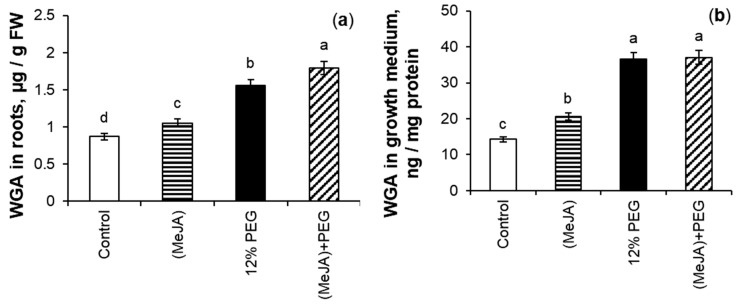
The effect of 10^−7^ M MeJA pretreatment for 24 h followed by 12% PEG exposure for 24 h on WGA accumulation in roots (**a**), and WGA excretion into the growth medium (**b**) of 8-day-old wheat seedlings. WGA—wheat germ agglutinin. Data are the mean ± SE (*n* = 9). Different letters above the columns imply that the values differ significantly at *p* < 0.05 (ANOVA, LSD test).

MeJA pretreatment stimulated WGA accumulation in wheat roots (Figure 9a) and its excretion into the growth medium (Figure 9b) by 21% and 44%, respectively, as compared to the control. Drought induced a 1.8-fold and 2.6-fold accumulation of this lectin in roots and nutrient medium, respectively, as compared to the control. During PEG exposure, MeJA pretreatment caused additional WGA accumulation in wheat roots by 15% as compared to the MeJA-untreated stressed wheat plants. MeJA pretreatment did not change WGA excretion level out of roots into the nutrition medium relative to MeJA-untreated stressed seedlings.

**Figure 10:**
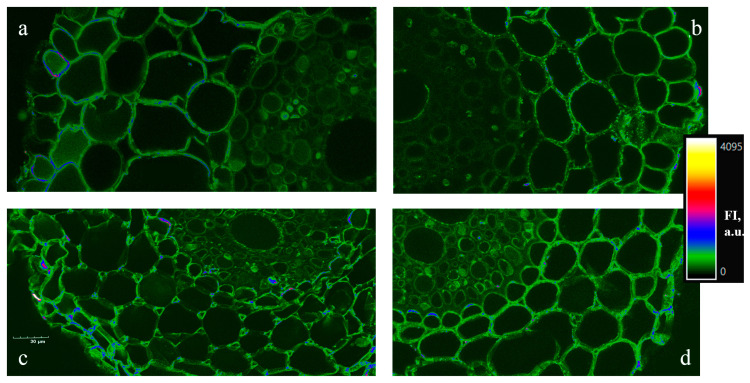
Cellular WGA localization in transverse sections of the proximal part of roots of 7-day-old wheat seedlings grown under 10^−7^ M MeJA pretreatment for 24 h and subsequent 12% PEG exposure for 5 h. (**a**)—control; (**b**)—(MeJA); (**c**)—12% PEG; (**d**)—(MeJA) + PEG. The bar on the right side of the image represents the WGA values converted into false color of fluorescence intensity (FI) scale. Bar scale = 30 µm.

Confocal microscopy was suitable for the purpose of specific localization of WGA protein in wheat roots and careful qualitative analysis. It showed WGA accumulation in the cell walls and cytosol near the cell walls of epidermis and exodermis of the control roots (Figure 10a). MeJA pretreatment caused a slight reduction in WGA deposition in these tissues (Figure 10b) as compared to the control. The greater intensity of WGA staining in the vascular cylinder was detected in plants exposed to 12% PEG, in comparison to the control (Figure 10c). During PEG exposure for 5 h, stress induced a significant fluorescence signal of WGA staining of cell walls between neighboring exodermal cells and U-shaped endodermal Casparian strips. During drought, MeJA pretreatment increased WGA accumulation in cell walls and cytosol near the cell walls of these tissues (Figure 10d) as compared to MeJA-untreated stressed plant roots.

### 2.7. Correlation Matrices

Under 10^−7^ M MeJA application for 0.5–2 h, the correlation coefficients for contents of ROS both in roots and in the growth medium, the activity of SOD, total POD, APX, and CAT of 6-day-old wheat roots were positive and equaled 1.

**Figure 11:**
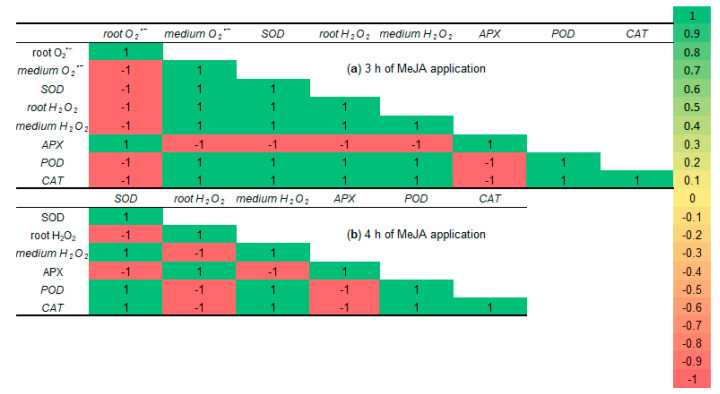
The correlation coefficient matrices for all examined parameters of 6-day-old wheat plants under 10^−7^ M MeJA application. The matrices are colored by magnitude and parity (green positive and red negative) for correlations at a *p* < 0.05 significance level.

After the third hour of MeJA application, the maximum negative correlations appeared (Figure 11a). The coefficient of O_2_^•−^ in wheat roots showed a strong negative correlation with all other parameters, except APX activity. This fact indicates a decrease in the trend of MeJA-induced superoxide anion generation. Moreover, APX activity also had significant negative correlations with all studied parameters, except O_2_^•−^ content in wheat roots. This fact is not surprising, as under the third hour of MeJA treatment, APX activity was near the control level.

During the fourth hour of MeJA application, SOD activity obtained the maximum negative correlation with APX activity and H_2_O_2_ accumulation in roots of wheat seedlings (Figure 11b). These facts may be explained by the different time of MeJA-induced activation of SOD and APX, and that H_2_O_2_ is not a substrate for SOD. APX activity had a significant negative correlation with parameters of SOD activity and hydrogen peroxide accumulation in the growth medium, but not with H_2_O_2_ content in the wheat roots. These facts prove short-term MeJA-induced activation of APX activity and different localization of APX (roots) and H_2_O_2_ (medium). As H_2_O_2_ is a substrate for APX, they have maximum positive correlation during 4 h of MeJA treatment. The maximum negative correlation coefficients for H_2_O_2_ in roots and all other studied parameters, except APX activity, point to a reduction in hydrogen peroxide content to control level, while H_2_O_2_ in the growth medium, SOD, and CAT activities were still over control during MeJA application for 4 h.

**Figure 12:**
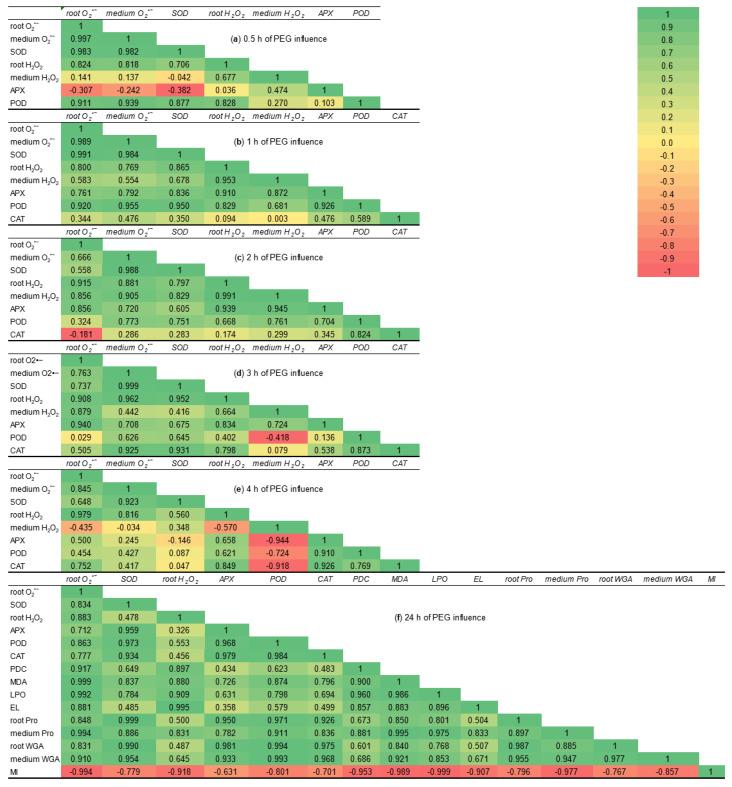
The correlation coefficient matrices for all examined parameters of 7–8-day-old wheat seedlings subjected to MeJA pretreatment and drought stress, caused by 12% PEG. The matrices are colored by magnitude and parity (green positive and red negative) for correlations at a *p* < 0.05 significance level.

During 0.5 h of stress subjection, the correlation coefficients can be grouped into three clusters: strong positive (from 0.7 to 1), weak positive (from 0.1 to 0.5), and negative (from −0.4 to −0.04) (Figure 12a). APX activity showed a negative correlation with SOD activity, O_2_^•−^ contents both in roots and in the growth medium. This fact indicates that during stress exposure for 0.5 h, the process of O_2_^•−^ generation/utilization and the induction of APX activity was at different time points. The H_2_O_2_ in the growth medium and SOD activity in the roots had a weak negative correlation, as they had different localization, and that hydrogen peroxide is not a substrate for the SOD enzyme. H_2_O_2_ in the growth medium had weak correlations with O_2_^•−^ both in roots and in the growth medium, due to the short time of stress influence. APX activity had a weak correlation with both H_2_O_2_ and total POD activity in roots because APX activated later than total POD.

During the first hour of PEG influence, the correlation coefficients of all studied parameters became positive (Figure 12b). The correlation coefficients between H_2_O_2_ in roots and both APX as well as total POD activities increased and reached the values 0.910 and 0.829, respectively, as compared to 0.5 h of stress exposure. The correlation coefficients between SOD activity, the levels of O_2_^•−^ both in roots and in the growth medium increased and became strongly positive, as they are components of one reaction. The correlation coefficients between H_2_O_2_ in the roots and SOD activity, O_2_^•−^ contents both in roots and in the growth medium varied from 0.769 to 0.865. The weakest, but still positive correlations had CAT activity with all studied parameters, their coefficients varied between 0.344 and 0.589. The correlation coefficients between CAT activity and hydrogen peroxide, both in wheat roots and the growth medium, were near zero due to later stress-induced CAT activation. During the 1 h of PEG exposure, the trend of correlation coefficients allowed us to assume the start of activation of the antioxidant enzymes’ defense of wheat roots.

During the second hour of PEG exposure, CAT activity still had a weak positive correlation with other studied parameters owing to the different time of their induction as compared to the control (Figure 12c). CAT activity and O_2_^•−^ accumulation in the roots of wheat seedlings had a negative correlation (−0.181), as O_2_^•−^ is not a substrate for CAT. The strong correlation of activities of oxidoreductase enzymes CAT and total POD (0.824) points to the induction of total POD and CAT activities under PEG influence for 2 h. Total POD activity had positive correlations with other studied parameters, their correlation coefficients varied from 0.668 to 0.773. There was only one exception: the correlation coefficient between total POD and level of O_2_^•−^ in the roots was weak, but positive (0.324), as O_2_^•−^ is not a substrate for POD. During the second hour of drought influence, the correlation coefficients between O_2_^•−^ content in the roots and O_2_^•−^ level in the growth medium (0.666) as well as SOD activity (0.558) decreased as compared to the first hour of PEG exposure. This fact points to the decrease in O_2_^•−^ generation and utilization during stress-induced oxidative burst. During PEG influence for 2 h, the correlation between H_2_O_2_ in roots, hydrogen peroxide in the medium and all studied parameters, except total POD and CAT activities, increased to high positive values (0.797–0.991) as compared to the first hour of PEG exposure due to the enhancement of H_2_O_2_ generation and utilization under drought-induced oxidative burst.

During the third hour of stress subjection, the correlation between total POD activity and H_2_O_2_ in the growth medium decreased to a negative value of −0.418 (Figure 12d) as these enzymes and ROS were spatially separated. The correlation coefficients between total POD activity and O_2_^•−^ content in roots as well as CAT activity and H_2_O_2_ in the medium were weak positive and reached 0.029 and 0.079 values, respectively. These findings point to the fact that superoxide anion is not the substrate for CAT enzyme, as well as the spatial separation of CAT (roots) and H_2_O_2_ (growth medium). The weak positive correlation coefficient (0.136) between APX and total POD indicates the different trends of activation of these oxidoreductases (Figure 6).

During the fourth hour of PEG exposure, H_2_O_2_ in the growth medium had strong negative correlation coefficients with APX, total POD, and CAT enzymes (Figure 12e). This fact can be explained by their different localization. H_2_O_2_ in the growth medium had negative correlation coefficients with both O_2_^•−^ (−0.435) and H_2_O_2_ in the roots (−0.570), as well as weak correlation with O_2_^•−^ in the medium (−0.034) due to the decrease of H_2_O_2_ exudation (Figure 5). The decrease of the correlation coefficients between APX, total POD, CAT and SOD activity in comparison to the third hour of stress influence points to a reduction of SOD activity (Figure 6).

During PEG subjection for 24 h, the most investigated parameters had changeable but positive correlation coefficients, except MI (Figure 12f), indicating that stress-induced regulation of plant growth and parameters of redox status are multidirectional processes.

## 3. Discussion

### 3.1. ROS Generation Under 10^−7^ M MeJA Application

The plant antioxidant system does not completely remove ROS under normal growth conditions (Figure 1 and Figure 3), [1] as ROS is involved in many developmental processes, including the maintenance of normal reproduction of crop plants [51]. The results of this work (Figure 1) and literature data [20,21,35,37,38] confirm the ability of jasmonates to induce both reactive nitrogen species and ROS accumulation in plant tissues. It is largely unclear how jasmonate treatment increases such signaling mediators, but it is known that NADPH oxidase (extracellular peroxidase) and polyamine oxidase (jasmonate signaling protein COI1) may be involved in jasmonate-induced generation of ROS wave in plants [52,53]. Transient trends of ROS accumulation in roots and the growth medium of wheat plants under MeJA treatment (Figure 1) demonstrated controlled character of MeJA-induced redox regulation during initial hours of phytohormone application and proposed their regulatory role in the jasmonate signaling pathway. Low and plant’s non-damaging concentrations of ROS control many processes in plant cells as downstream components of the JA-dependent signal mediator [37], including ROS involvement in the induction of jasmonate synthesis in plant tissues [21].

### 3.2. SOD, Total POD, APX, and CAT Activities During MeJA Treatment

Jasmonates can regulate the ROS-generating enzymes’ activity [21]. MeJA-induced transient ROS accumulation (Figure 1) may act as a signal and a component of the redox system, activating the plant antioxidant system [20,21]. In this work, MeJA-induced changes in the O_2_^•−^ and H_2_O_2_ levels in the wheat roots and growth medium (Figure 1) correlated with the transient increase in activity of antioxidant enzymes SOD, CAT, total POD, and APX in wheat roots (Figure 2 and Figure 3). It is interesting to notice that MeJA induced activation of peroxidases (APX and total POD) and SOD enzymes earlier than CAT. Literature data confirm jasmonates’ ability to stimulate enzymatic and non-enzymatic antioxidant activity of different plant species [20,37], including rice [54], as compared to the control plants. The 10^−7^ M MeJA-induced regulation of O_2_^•−^ and H_2_O_2_ accumulation, SOD, total POD, APX, and CAT activities in wheat roots indicates ROS involvement in MeJA signaling. Using coefficients of enzymes’ activity/ROS, it was possible to identify the ability of MeJA treatment to regulate the redox status of wheat roots and correctly interpret the obtained results (Figure 3). No significant MeJA influence on peroxidase activities was found (Figure 3b,c). The obtained results indicate MeJA involvement in the regulation of SOD (Figure 3a) and CAT activity (Figure 3d).

### 3.3. The Effect of MeJA Pretreatment on MI and PDC Under 12% PEG Exposure

Drought is one of the major environmental stress factors that restricts wheat plants’ growth parameters. Jasmonates had a favorable influence on the growth of a variety of species under different abiotic stresses [31,32,33,34]. The results of this work showed that during drought MeJA pretreatment increased the division of cells of wheat root tips as compared to MeJA-untreated stressed seedlings (Figure 4b). Such MeJA-induced effect on the growth of wheat plants may be achieved by preventing sharp stress-induced changes in plant hormonal system [36,55] and accumulating “growth-inducing” phytohormones [14]. The MeJA application is able to induce genes related to photosynthesis gene expression, improve the photosynthetic efficiency of leaves, which in turn has a beneficial effect on plant growth processes [56]. It is known that jasmonates and stress-induced oxidative burst can regulate the cell cycle of plants [19,57]. ABA-induced ROS accumulation in root tips of *A. thaliana* seedlings is an important signal regulating the division activity of meristematic cells [58], and such regulation may also occur during jasmonate application.

The plasma membranes of the living cells prevent the penetration of big dye molecules of Evans blue into the cytoplasm, so more than 60% of root cells remained unstained (Figure 4a). Cells are considered intact when the percentage of damaged cells in tissues is less than 50% [59]. The results of this work point out that MeJA-induced transient ROS generation (Figure 1) may be attributed to changes in signaling, as the absence of damaging effect on the division of meristematic cells of wheat root tips and a decrease in the PDC level were detected (Figure 4). After stress-induced damage to wheat root membranes, the dye stained the cytoplasm, and the amount of dye entering the cells corresponds to the degree of damage to tissue cells, caused by 12% PEG exposure. It should be noted that cell damage also occurs during normal plant ontogenesis, for example, during the initiation of lateral roots, etc. MeJA pretreatment mitigated membrane permeability for dye both under non-challenging growth conditions and drought (Figure 8).

### 3.4. The Effect of MeJA Pretreatment on ROS Generation in Roots Under 12% PEG Exposure

The MeJA application transiently increased ROS production in roots and Hoagland–Arnon solution (Figure 1) under non-challenging growth conditions. It is likely that ROS, as secondary mediators, are involved in jasmonate-induced preadaptation of neighboring plants to abiotic stresses [21]. Under 12% PEG exposure, MeJA pretreatment significantly declined stress-induced ROS generation in wheat roots and in the growth medium (Figure 5, Table 1) as compared to MeJA-untreated stressed seedlings. The literature data confirm the ability of jasmonate treatment to reduce ROS accumulation in plants during abiotic stresses [28,33,56]. It could be assumed that H_2_O_2,_ as a secondary ROS signal [60], was attenuated due to MeJA pretreatment. Under PEG influence, the MeJA-induced reduction in ROS generation may be the reason for the enhancement of MI of wheat root tips and the decrease in PDC level as compared to MeJA-untreated stressed seedlings.

### 3.5. MeJA-Induced Regulation of Activity of Antioxidant Enzymes Under Water Deficit

Antioxidant enzymes contribute to the process of tight control between ROS production and scavenging. During the first days of drought, stress significantly and transiently increased SOD, total POD, APX, and CAT activities in roots of wheat seedlings (Figure 6, Table 1) as compared to the control. During 12% PEG exposure, MeJA pretreatment stimulates a further induction of SOD, total POD, APX, and especially—CAT activities in wheat roots in comparison to MeJA-untreated stressed seedlings. SOD activity showed such additional stress-induced stimulation earlier than total POD, APX, and CAT ones. The results of this work demonstrate MeJA’s ability to regulate the activity of antioxidant enzymes and induce the protection of root growth (Figure 4) under stressful conditions.

It is known that MeJA-induced oxidative burst alleviation and plant stress resistance occurred as a result of increased antioxidant capacity [28,33,34]. Literature data prove jasmonates’ ability to induce additional activity of ROS-scavenging enzymes of a variety of plant species under long-term abiotic stress influences [31,57,61,62]. This effect might be achieved by the collaborative action of jasmonates and other growth regulators. For example, during drought, the combined MeJA and salicylic acid pretreatment decreased lipid peroxidation and H_2_O_2_ generation in maize due to an increase in proline accumulation and the activities of CAT, POD, and SOD [63]. In this investigation, during PEG exposure for 24 h, MeJA could increase the activity of SOD, total POD, APX, and CAT enzymes (Figure 6 and Table 1), and thus this phytohormone may reduce the development of oxidative burst. It is important to highlight the fact that CAT is the most sensitive enzyme to MeJA regulation.

### 3.6. MeJA-Induced Mitigation of MDA During Drought Stress

The estimation of EL and MDA parameters revealed the MeJA ability to decrease stress-induced damages in membranes of wheat roots (Figure 8, Table 3). MDA concentration and localization were determined by biochemical method (Table 3) and by staining roots with Schiff’s reagent (Figure 8). MeJA pretreatment did not change the lipid peroxidation in the tips of wheat roots and the whole root system, while it slightly increased EL under optimal growth conditions. The EL enhancement may indicate an increase in the process of exudation by the roots. During PEG exposure, MeJA pretreatment decreased the levels of MDA and EL of wheat roots as compared to MeJA-untreated stressed seedlings. The results received in this work are consistent with literature data obtained from different plant species and under exposure to a wide range of abiotic stresses [26,31,40].

### 3.7. MeJA-Induced Regulation of Pro and WGA Accumulation Under Short-Time Water Deficit Stress

During stress conditions, plants increase the diversity of metabolites that can provide protection to plants from stress factors [14]. One of the plants’ mechanisms to tolerate water deficit is the production of secondary metabolites with antioxidant capacity and other useful properties for plant tolerance, which allow plants to mitigate environmental stress [1]. Proline is a well-known, very soluble in water amino acid and a compatible osmolyte. Pro has high hydrophilicity, participates as a signal and/or regulatory molecule in signal pathways, may be the ROS scavenger, contributor to osmotic adjustment, up-regulator of enzymatic antioxidants [64], regulator of cell osmotic pressure, and safeguard of cell macromolecule structures’ stability [65]. Pro accumulation in plant tissues increased during stresses, including osmotic stress [6,35] (Table 3). Sirhindi et al. (2016) found that during the influence of heavy metals, jasmonates caused an additional increase in Pro level in soybean plants [31]. Under 12% PEG exposure, MeJA pretreatment induced additional Pro generation in wheat roots and declined its accumulation in the growth medium as compared to MeJA-untreated stressed seedlings. The results demonstrated the importance of Pro accumulation precisely in plant organisms, but did not exclude the signal and protective roles of this amino acid in the nutrition medium. It is known that plants increase Pro exudation under drought conditions [66]. The level of exogenous Pro can be used for the detection of plant stress [11]. Exogenous Pro acts as a signal, provides beneficial effects on growth, and induces stress tolerance in many plant species [64,67]. Proline exposure to plants mitigated abiotic stress influence due to its ability to enhance the activity of antioxidant enzymes [55,68] and ROS scavenging [69], influence on water balance, DNA stability [70], and delaying plant wilting [71].

Drought significantly increases soluble protein content in plant tissues, protecting the stability of cell membrane structures to maintain normal function to ensure optimal crop growth [72]. Plant lectins are universal and sensitive recognition systems on the cell’s surface, facilitating even the detection of symbiotic and pathogenic organisms [45]. The involvement of plant lectins in monitoring cell wall structure and cell growth is also discussed. WGA can interact with plant growth-promoting bacteria, *Bacillus subtilis,* and stimulate the growth of bacterial colonies [73]. WGA pretreatment also had the growth-stimulating and protective effect on the MI of meristematic cells of roots of monocotyledonous plants barley and rice under salinity [48]. The quantitative analysis of WGA level obtained by ELISA demonstrates that water deficit, modulated by 12% PEG, induced significant WGA accumulation in root tissues (Figure 9a and Figure 10) and increased WGA content in the nutrition medium of wheat seedlings (Figure 9b). Under drought, MeJA pretreatment additionally increased WGA accumulation in roots, but did not change the WGA level in the growth medium as compared with MeJA-untreated stressed plants. This fact highlights the importance of WGA within plant organisms and allows us to suggest WGA involvement in signal transduction during stress influence and/or phytohormone treatment. Since both amino acid and protein synthesis are very energy-intensive processes, under stress conditions, it should be useful for plants to accumulate them in the tissues rather than excrete them into the environment. It should be noted that WGA protein contains a number of antioxidant polypeptides [74], so wheat lectin could be a part of the antioxidant system.

Understanding the localization of defense proteins within the cells, as well as their amount, is essential for proteomics research. WGA is widely present in wheat root tissues, and WGA protein assay was conducted in the transverse sections of the proximal part of 7-day-old wheat roots (Figure 10). The mature cells in that part of the root contain a big vacuole, so the thin layer of cytoplasm is pinned down to the cell wall. Due to the proposed WGA functions as an excreted and defense protein, WGA was preferentially localized in cells of epidermis and exoderm of the control and MeJA-pretreated wheat roots. The predominant WGA localization in the peripheral part of roots (rhizoderm and exoderm) (Figure 10a,b) suggests that this exuded lectin has some structural, signaling, and/or protective function. Weak staining of the vascular cylinder tissues proves this suggestion. 12% PEG exposure for 5 h reduced the cell size of roots, probably owing to cell dehydration. It can be assumed that under water deficit, the increase in staining intensity in the vascular cylinder may be due to stimulation of the lectin’s transport from the site of synthesis (roots). It is interesting to note that under PEG exposure, the images showed the specific increase in WGA staining of membranes and cytoplasm of exodermal cells, which in conjunction with neighboring cells and cavities of parenchyma cells. During stress influence, MeJA pretreatment induced WGA deposition in all root tissues as compared to MeJA-untreated stressed seedlings. The negative control without primary antibodies to WGA showed the absence of staining. It can be assumed that WGA is involved in the regulation of intercellular interaction under water deficit. During PEG exposure, the loose parenchyma in the exoderm with developed intercellular spaces may serve for oxygen and water storage, alleviation of the excretion of different substances, including WGA and Pro, into the root surrounding medium.

Under abiotic stress influence, the regulation of proline and lectin accumulation in plants is complex and depends on a variety of external and internal signals. Apart from ABA, other factors are involved in the regulation of WGA gene expression and Pro accumulation during stress influence [64,75,76]. MeJA may also regulate Pro and WGA accumulation in roots of wheat seedlings and their excretion into the nutrition medium [31,35] (Table 3, and Figure 9 and Figure 10) under non-challenging growth conditions and drought influence.

## 4. Materials and Methods

### 4.1. Plant Material and Treatments

Winter wheat seeds (*Triticum aestivum* L.) of cultivar Scepter obtained from Chishminsky Crop Production Station, Russia, were used as the object of research. The seeds were surface sterilized with 96% ethanol, washed, sown in Petri plates containing wet filter paper, and then grown for 6 days on 10% Hoagland–Arnon solution. The solution changed every day. Wheat seedlings were grown in a climatic chamber under illumination of 200 mmol m^−2^s^−1^ at 22–24 °C and 16 h photoperiod.

The wheat seedlings at the water-sensitive first leaf stage were used in this investigation. The roots of 6-day-old wheat seedlings were treated with 10^−7^ M MeJA for 0.25–5 and 24 h by adding the phytohormone to the nutrient medium. It is known that MeJA at a concentration of 10^−7^ M has a growth-stimulating effect on wheat plants [35]. The control plants were grown in a 10% Hoagland–Arnon solution. On the seventh day, the MeJA solution was removed, and some of the seedlings were subjected to short-term (0.5–5 and 24 h) drought stress, modulated by 12% PEG 6000 (PanReac AppliChem, Barcelona, Spain) in 10% Hoagland–Arnon solution. The plant’s physiological responses to such a high concentration of PEG mimicked drought due to the difficulty in the uptake of water by the plants. The rest of the plants continued to grow in a 10% Hoagland–Arnon nutrient solution. The roots of 7- and 8-day-old wheat seedlings were analyzed for different parameters. The levels of prooxidants, WGA, and Pro were determined in the roots and in the incubation medium of wheat plants. The activities of SOD, APX, total POD, CAT, the parameters of EL, and MDA were defined in the roots. The detailed experimental operations could be found in previous work [77].

### 4.2. Assay of Antioxidant Enzymes

Superoxide dismutase (SOD, EC 1.15.1.1) activity was measured in wells of a flat-bottomed plate for immunoassay (Costar, Washington, DC, USA) by spectrophotometer EnSpire 2300 (Perkin Elmer, Waltham, MA, USA) at 540 nm according to Beyer and Fridovich [78]. One unit of the SOD activity (U) was defined as the amount of enzyme required to result in a 50% inhibition of the rate of nitro blue tetrazolium reduction at 540 nm.

Catalase (CAT, EC 1.11.1.6) activity was determined by measuring the decomposition of H_2_O_2_ by a Benchmark microplate reader (Bio-Rad, Hercules, CA, USA) at 405 nm, as described by Aebi [79].

Ascorbate peroxidase (APX, EC: 1.11.1.11) activity was assayed as described by Verma and Dubey [80] in wells of a flat-bottomed plate for immunoassay (Costar, USA) at 290 nm.

Total peroxidase (POD, EC: 1.11.1.7) activity was assayed as described by Yusupova et al. [81] by scanning the ability of enzyme extracts to prevent *o*-phenylenediamine (OPD) oxidation. The roots of 5 seedlings were homogenized in 0.01 M potassium phosphate buffer at pH 6.0 based on the ratio 1:10 (*m*/*v*) and extracted for 30 min at 4 °C. The extracts were centrifuged at 13,000× *g* for 10 min at 4 °C, and the activity was assayed by a micro method in wells of a flat-bottomed plate for immunoassay (Costar, USA). The reaction was initiated by the addition of the plant extract for 1 min, followed by reaction termination by the addition of 4 N H_2_SO_4_. Samples were measured by a Benchmark microplate reader (Bio-Rad, Hercules, CA, USA) at 492 nm. Results were given as a specific activity Units/(mg protein × min).

Total protein content in the extracts was quantified by the Bradford method [82] in wells of a flat-bottomed plate for immunoassay (Costar, USA), with bovine serum albumin (MilliporeSigma, Darmstadt, Germany) as the standard, by spectrophotometer EnSpire 2300 (Perkin Elmer, Waltham, MA, USA) at 595 nm.

### 4.3. Determination of Superoxide Anion Radical in the Plant Growth Medium

Extracellular production of O_2_^•−^ was measured by oxidation of epinephrine (ICN, New York, NY, USA) to adrenochrome [83] by spectrophotometer Smart Spec Plus (Bio-Rad, Hercules, CA, USA) at 490 nm and expressed as nM O_2_^•−^/(g FW × min).

### 4.4. Estimation of O_2_^•−^ Content in Roots of Wheat Seedlings

The level of O_2_^•−^ in root tissues was determined based on the method proposed by Chaitanya and Naithani [84]. The superoxide anion concentration was measured by its ability to reduce nitro blue tetrazolium (Merck, Darmstadt, Germany). The absorbance of the reaction mixture was measured after 1 min at 540 nm using a spectrophotometer Smart Spec Plus (Bio-Rad, Hercules, CA, USA).

### 4.5. Extracellular H_2_O_2_ Content

The content of H_2_O_2_ in the plant growth medium was determined by oxidation of OPD with hydrogen peroxide according to Khairullin et al. [85] by Benchmark microplate reader (Bio-Rad, Hercules, CA, USA) at 492 nm.

### 4.6. Estimation of H_2_O_2_ Content in Roots of Wheat Seedlings

H_2_O_2_ content was determined in the root extracts by the method described by Bellincampi et al. [86] by spectrophotometer EnSpire 2300 (Perkin Elmer, Waltham, MA, USA) at 570 nm. H_2_O_2_ content was calculated using a standard curve based on the absorbance of H_2_O_2_ standards.

### 4.7. Malondialdehyde (MDA) Accumulation in Roots of Wheat Seedlings

MDA, the main metabolite of lipid peroxidation, was determined by measuring its reaction with the thiobarbituric acid, following the modified method of Heath and Packer [87] at 532 nm and 600 nm using a spectrophotometer Smart Spec Plus (Bio-Rad, Hercules, CA, USA). MDA content was calculated using the molar extinction coefficient of 155 mM^−1^cm^−1^.

### 4.8. Estimation of Electrolyte Leakage (EL)

After the plants were incubated in a glass jar containing 20 mL distilled water with shaking at 130 rpm, 30 °C for 2 h by orbital shaker-incubator ES-20 (Biosan, Riga, Latvia) [88], the electrical conductivity of the incubation solution was recorded by portable multi-range conductivity/TDS meter HI 8633 (HANNA instruments, Veneto, Italy).

### 4.9. Proline (Pro) Accumulation in Roots and in the Plant Growth Medium

A total of 0.5 g of fresh root material was soaked in 3 mL of hot distilled water and boiled in a water bath for 1 h at 100 °C [89]. The immediately cooled extract (1 mL) or 1 mL of 20 wheat plants growth solution was mixed with 1 mL of glacial acetic acid-ninhydrin reagent and 1 mL of glacial acetic acid in a test tube, and the mixture was placed in a water bath for 1 h at 100 °C. The absorbance of the cold reaction mixture was measured at 522 nm using a spectrophotometer Smart Spec Plus (Bio-Rad, Hercules, CA, USA). The Pro was calculated by comparison to a standard curve of proline; the results were expressed as pM Pro/g FW (for proline determination in the growth medium) or µM Pro/g FW (for proline determination in roots) [90].

### 4.10. Analysis of Percentage of Death Cells (PDC) of Wheat Roots

The percentage of dead cells was analyzed by the Evans blue method [88] at 600 nm using a spectrophotometer Smart Spec Plus (Bio-Rad, Hercules, CA, USA); the results of cell death in roots were given as a percentage of frozen and thawed roots.

### 4.11. Histochemical Detection of Lipid Peroxidation

The lipid peroxidation of the root tips was determined histochemically by the Schiff’s reagent (Merck, Darmstadt, Germany) according to [91]. The red/purple color indicated the localization of aldehydes formed during peroxidation was monitored using a Biozero microscope (Keyence, Osaka, Japan) at 4×. Then, the images were analyzed using Image J 1.41o/Java 1.6.0_10 software (NIH, Bethesda, MD, USA) based on the integrated density multiplied by painted area to obtain relative total painted values [92]. For better presentation of results, relative levels of lipid peroxidation were divided by 10^10^.

### 4.12. Mitotic Index (MI) Analysis

MI was determined from changes in activity of the division of the apical meristem cells of wheat seedling roots [50] by a light Biozero microscope (Keyence, Osaka, Japan) at a magnification of 40×. Each variant of the experiment included no less than 20 seedlings. MI was calculated as a percentage of the dividing cells.

### 4.13. Indirect Immunohistochemical Localization of Wheat Germ Agglutinin (WGA) Using Confocal Microscopy

The freshly cut off of basal part of wheat roots (1 cm after the site of the first future tillering node) of 4 mm in length were fixed and embedded in solution buffer (SB), containing 12.5 _M_M PIPES for buffer solutions (PanReac, Castellar del Vallès, Spain), 1.25 _M_M MgSO_4_, 1.25 _M_M EGTA (Sigma-Aldrich, Saint Louis, MO, USA), 3% paraformaldehyde (PanReac, Spain), 0.25% glutaraldehyde (PanReac, Spain), 0.3% Tween-20 (Thermo Scientific, Waltham, MA, USA), 0.3% Triton-X-100 (Serva, Heidelberg, Germany), DMSO (Merck, Germany), pH 6.9 during 16 h at 4 °C according to [93]. Root sections embedded in 3% agarose LE2 (Helicon, Moscow, Russia), which is used as support to mount and section plant materials to slices 45 µm thick with a sapphire knife on a vibrotome (Leica VT 1200 S, Wetzlar, Germany). To exclude nonspecific staining, sections were treated for 2 h at room temperature with a blocking solution containing 12.5 mM PIPES, 1.25 mM MgSO_4_, 1.25 mM EGTA, 0.2% gelatin (Merck, Germany), and 5% bovine serum albumin (BSA, Sigma-Aldrich, Saint Louis, MO, USA). Transverse sections of the proximal part of wheat roots were then stained for WGA localization by incubating sections with primary polyclonal antiWGA rabbit antibodies (1/2000, *v*/*v*), obtained in our laboratory, for four days at 4 °C in SB with 1% BSA. Then sections were washed 6 times for 30 min in SB with 1% BSA, 0.1% Tween-20, 0.1% Triton-X-100, and stained with goat anti-rabbit second antibodies, labeled with fluorescent tag Alexa Fluor 488 (1:2000, *v*/*v*) (ab_2556544, ThermoFisher, Waltham, MA, USA) by incubation in SB with 1% BSA for 2 h at dark and room temperature. Pro Long Gold antifade reagent (Life Technologies Corporation, Eugene, OR, USA) for 16 h in the dark and at room temperature was used for placing the sections under a cover glass. Fluorescence signals of WGA-stained lectins were detected using a confocal laser scanning microscope (Olympus FV 3000, Osaka, Japan) at 40× magnification, and monitored with the laser with an excitation line of 488 nm and an emission window from 500 to 550 nm. For each treatment, at least 10 sections of 3 plants were photographed using the Olympus FV 3000 compound microscope, and the representative photo was chosen for each treatment. The normalized signal of fluorescence intensity (FI) of WGA is visualized as a color spectrum.

### 4.14. Estimation of WGA Accumulation in Roots and in the Nutrition Medium

The content of WGA was measured in excised roots of 10 seedlings (nearly 0.5 g FW) and in their growth medium by ELISA as described by Shakirova et al. [94,95]. Wheat seedling roots were homogenized in 0.05 M HCl for 1 h at room temperature and centrifuged with 6000× *g* for 20 min. Supernatants were neutralized with 1 M sodium phosphate buffer (pH 7.2). Neutralized supernatant and growth medium solution were tested for WGA content by immunoassay [96] using a flat-bottomed plate for immunoassay (Costar, USA). Absorption of the samples was measured by a Benchmark microplate reader (Bio-Rad, Hercules, CA, USA) at 492 nm. WGA content in roots was expressed as mg WGA/g FW, while WGA level in the plant growth medium was expressed as ng WGA/mg protein.

### 4.15. Statistical Analysis

All experiments were performed in triplicate, and the experimental data were subjected to a one-way analysis of variance (ANOVA) using the SPSS 19.0 software (SPSS Inc., Chicago, IL, USA). Significant differences between values were determined using the LSD test at *p* < 0.05. Data in the figures are presented as mean values and their standard errors (±SE).

## 5. Conclusions

To manage drought, different tools are used to enhance crop tolerance under drought stress conditions. In this study, 10^−7^ M MeJA treatment was able to complex and time-dependent mitigation of osmotic stress damage in wheat roots, modulated by 12% PEG. This work revealed MeJA’s ability to reduce drought-induced oxidative stress by increasing the activity of enzymatic antioxidants in wheat roots. The results presented in this article, which used MeJA as a model, demonstrate the huge potential for jasmonate application in agriculture and indicate the need for further research on this problem. The benefits of applying jasmonate phytohormones can help develop plant varieties that are resistant to environmental stresses and generally efficient agricultural systems. MeJA’s ability to control ROS production sheds light on the dual role of jasmonates in plant signaling and regulation of oxidative stress under water deficit stress. This regulation depends on different circumstances, such as cultivar [6,10,15], concentration of jasmonate in the preparation used [15,16,56], method and time of processing [24], and strength of stress exposure. The regulation of plants’ antioxidant system under the influence of environmental factors may be complex and can involve ABA [39], salicylic acid, NO, and H_2_O_2_ [13].

Except for Pro and NO [77], WGA is another compound with potential signaling properties. The immunohistochemical and biochemical observations reinforced the previously obtained results concerning the ability of WGA to reduce the level of oxidative stress in wheat plants under salinity [50]. So, amino acid Pro and lectin WGA are likely involved in the signaling and complex regulation of stress tolerance of wheat plant roots, including their redox status. The mechanisms of how Pro and WGA protect plants against drought are not fully understood, and they should be elucidated. Focusing the researchers’ attention on the effects of functional interactions between jasmonates, non-protein signaling mediators ROS, and amino acid Pro and protein WGA in the wheat organism will create opportunities for a better understanding of jasmonates protective effects under abiotic stresses [21].

## Figures and Tables

**Table 1:** The effect of 10^−7^ M MeJA pretreatment for 24 h followed by 12% PEG exposure for 24 h on levels O_2_^•−^, H_2_O_2_, SOD, total POD, APX, and CAT activities in roots of 8-day-old wheat seedlings.

Treatment	O_2_^•−^ in Roots, OD_540_/ (g FW × min)	H_2_O_2_ in Roots, µM/g FW	SOD Activity, U/(mg Protein × Min)	POD Activity, U/(mg Protein × Min)	APX Activity, µM Ascorbate/ (mg Protein × Min)	CAT Activity, µM H_2_O_2_/(mg Protein × Min)
Control	0.30 ± 0.015 c	153.8 ± 6.9 b	90.6 ± 4.1 c	11.1 ± 0.5 b	1.8 ± 0.09 d	121.7 ± 5.9 d
(MeJA)	0.29 ± 0.015 c	148.3 ± 7.1 b	92.4 ± 4.2 c	12.3 ± 0.6 b	2.3 ± 0.11 c	182.7 ± 8.1 c
12% PEG	0.53 ± 0.026 a	240.0 ± 10.5 a	121.5 ± 6.1 b	14.9 ± 0.7 a	2.8 ± 0.14 b	237.0 ± 10.6 b
(MeJA) + PEG	0.44 ± 0.022 b	164.0 ± 7.8 b	135.2 ± 6.5 a	15.6 ± 0.7 a	3.5 ± 0.17 a	268.0 ± 11.4 a

Values are the mean of three replicates ± SE (*n* = 12). Different letters show a significant difference at *p* < 0.05.

**Table 2:** The effect of 10^−7^ M MeJA pretreatment for 24 h followed by 12% PEG exposure for 24 h on coefficients of SOD activity/superoxide anion, POD activity/hydrogen peroxide, APX activity/hydrogen peroxide, and CAT activity/hydrogen peroxide of roots of 8-day-old wheat seedlings.

Treatment	SOD Activity/O_2_^•−^	POD Activity/H_2_O_2_	APX Activity/H_2_O_2_	CAT Activity/H_2_O_2_
Control	302 ± 15 a	0.072 ± 0.003 c	0.012 ± 0.0006 c	0.791 ± 0.04 d
(MeJA)	319 ± 16 a	0.083 ± 0.004 b	0.016 ± 0.0008 b	1.232 ± 0.06 b
12% PEG	229 ± 10 b	0.062 ± 0.003 d	0.012 ± 0.0006 c	0.988 ± 0.05 c
(MeJA) + PEG	307 ± 15 a	0.095 ± 0.005 a	0.021 ± 0.0010 a	1.634 ± 0.08 a

Values are the mean of three replicates ± SE. Different letters show a significant difference at *p* < 0.05.

**Table 3:** The effect of 10^−7^ M MeJA pretreatment for 24 h followed by 12% PEG exposure for 24 h on levels of MDA, electrolyte leakage of roots, Pro accumulation in roots and in the growth medium of 8-day-old wheat seedlings.

Treatment	MDA, nM/g FW	EL, mS/g FW	Pro in Roots, µM/g FW	Pro in the Growth Medium, pM/g FW
Control	22.45 ± 0.55 c	22.97 ± 0.55 d	0.80 ± 0.04 c	0.30 ± 0.009 d
(MeJA)	22.66 ± 0.56 c	27.07 ± 0.65 c	0.82 ± 0.04 c	0.37 ± 0.010 c
12% PEG	42.72 ± 0.98 a	92.11 ± 2.10 a	1.80 ± 0.09 b	17.73 ± 0.43 b
(MeJA) + PEG	35.04 ± 0.86 b	35.00 ± 0.85 b	2.20 ± 0.10 a	12.90 ± 0.32 a

MDA—malondialdehyde; EL—electrolyte leakage; Pro—proline. Values are the mean of three replicates ± SE. For MDA determination, *n* = 6, for proline content and EL estimation, *n* = 9. Different letters show a significant difference at *p* ≤ 0.05.

## Data Availability

Data are contained within the article.
